# Bibliography

**Published:** 1900-12

**Authors:** 


### Bibliography.

Manual of Pathology, including Bacteriology, the Technic of Post-Mortems, and Methods of Pathologic Research. By W. M. Late Coplin, M.D., Professor of Pathology and Bacteriology, Jefferson Medical College, Philadelphia; Pathologist to Jefferson Medical College Hospital, etc., etc. Third Edition, revised and enlarged. With Three Hundred and Thirty Illustrations and Seven Colored Plates. P. Blakiston's Son & Co., Philadelphia, 1900.

This, the third edition of this valuable work, is, as the author states in the preface, not to make it "a treatise or book of reference, but, as its title indicates, a manual that the author hopes may be useful in the laboratory and post-mortem room and in clinical diagnosis by the aid of the microscope." This altogether too modest idea of a book of eight hundred and forty-six pages does not fully give the reviewer's conception of the work. While it is true that it will not satisfy the general reader, in that he will fail to find much there regarded as a necessity in a book of reference, he will, on the other hand, have no cause to complain of the want of lucidity in the descriptions.

Following out the general plan of the book, that of a practical assistant to the study of pathology, the author naturally opens the first chapter with "Technic, Post-Mortem Examinations, Instruments needed." These are fully covered and the necessary instruments carefully illustrated. Following this chapter, in proper order, comes the "Technic of Morbid Histology." This, together with "Bacteriological Technic," is satisfactorily illustrated. The "Illustrations of Various Sediments" is very complete, and should be of great value to the student in differentiating the various presentations under the microscope. The "Technic of Sputum Examinations" follows in regular order, and this closes Part I. and forms the basis for General Pathology in Part II.

The author prefaces this part of the subject by a discussion as to the "Causes of Disease." This is a very clear statement, and is one of the most interesting chapters in the book, dealing, as it does, with all those changes that lead to abnormality in tissue, and finally to the discussion of immunity to disease, together with the theories connected with this. That absolute immunity rarely exists is a statement that possibly cannot be controverted, for immunity when apparently well assured may be broken down by various methods. The author has given much space to "Bacteria as Causes of Disease." Nothing more fully illustrates the change in the study of Pathology than this extended consideration. The pathological books of a very few years ago gave this but slight attention, and it is within the memory of many when it was practically unknown. It is due to this advanced knowledge that pathological studies have become truly scientific and have raised the general practice of medicine from the empirical stage which it occupied at the early part of the last half of this century. The subject is treated here as fully as could be expected, and this and the following related subject, that of animal parasites, is very satisfactory.

Chapter II. deals with "Hypertrophy, Hyperplasia, Metaplasia, Heteroplasia, Atrophy, Hypoplasia, Agenesis, or Aplasia." This is followed by Chapter III. on "Infiltration and Degeneration," and Chapter IV. on "Necrosis," and Chapter V. on "Circulatory Disturbances;" and then the author opens Chapter VI. with "Inflammation and Repair." He accepts Park's definition of inflammation, which is that given in "Park's Surgery,"—"Inflammation is an expression of the effort made by a given organism to rid itself of or to render inert noxious irritants arising from within or introduced from without." The reviewer is inclined to agree with Stengel that no short definition contains the essence, for inflammation is by no means a simple process. If a definition must be given, that of Burdon Sanderson seems preferable, that inflammation is the "Succession of changes which occur in a living tissue when it is injured, provided that the injury is not of such degree as at once to destroy its structure and vitality." This chapter covers all there is of a practical nature in this condition, and is so far very satisfactory; but the critical reader misses some of the work of many investigators, and he will search in vain for any allusions to the earlier history of this subject. While this may be dead material, the living student cannot appreciate the present advanced position it occupies unless he is made familiar with the work that has preceded this era. It is understood that this could not be expected in a manual, and therefore, while the loss is recognized, it is not stated as a criticism.

The author seems to regard the diapedesis of the leucocyte as the principal origin of pus, although he acknowledges that proliferation may have a certain influence in the production of pus-cells. This older theory of Virchow, so strenuously combated by Conheim and his school, seems not to have lost ground by time, as all pathologists recognize the possibility of two sources of pus, —migration and proliferation.

Space will not permit an extended review of all the chapters of this interesting production, but allusion must be made to Chapter XV., on "Bones and Joints," and to the sub-chapter, "Caries and Necrosis." In this it is stated that "Death of the bone and the process of separation from the living tissue are comparable to the changes already noted as present in soft parts. Where the dead tissue joins the living there appear accumulations of phagocytic cells that attack the osseous matrix forming the line between the dead and living structures and proceed with the separation of the necrotic mass. Many of these cells are clearly leucocytes belonging to the phagocytes already described. Other cells resemble the giant cells." This lucid explanation is far in advance of that usually given in the unmeaning phrase, "That a line of demarcation is formed," etc., without a word as to the cause which separates the dead from the living bone.

While the book will, naturally, appeal more to students of general pathology, those who aim to be stomatologists in fact rather than in name cannot narrow their studies to special pathology; in fact, in this instance the greater includes the less in its enunciation of foundation principles, and without a knowledge of the former the latter cannot be understood. With Dr. Coplin's book as a guide they cannot go far astray, but will follow his teaching with interest and profit.

This book is produced in the usual excellent style of the publishers. The illustrations and colored plates, many of which are original, give additional satisfaction to the reader, and especially are they of value to students, leading to a better comprehension of the subject.

DENTAL METALLURGY. A MANUAL FOR THE USE OF DENTAL STUDENTS AND PRACTITIONERS. By Charles J. Essig, M.D., D.D.S., Professor of Mechanical Dentistry and Metallurgy in the Dental Department of the University of Pennsylvania. Revised by Augustus Koenig, B.S., M.D., Demonstrator of Metallurgy in the Dental Department of the University of Pennsylvania, etc. Fourth Edition, revised and enlarged. Illustrated with Forty-three Engravings. Lea Brothers & Co., Philadelphia and New York.

There is no better evidence of the practical value of a book than repeated editions. It is no test, however, of its scientific value, for it is a notorious fact that the nearer a scientific book approaches originality the slower will be the sale. This is readily accounted for from the fact that the circle of readers must necessarily be limited.

To prepare a book to meet alike the needs of undergraduates and practitioners requires a judicious handling of subjects, the tendency frequently being to write beyond the mental capacity of the one, or, on the other hand, giving too much attention to detail to satisfy the other. It is thought the author of this book has, as a rule, happily avoided this difficulty and given the dental profession a work that will enable both young and old to grasp the subjects upon which it treats.

When this book first appeared it was in advance of the series of dental text-books that have since been issued prolifically from the press. Indeed, it was the first ever issued upon metallurgy in the form of a manual for dental students and practitioners. It was presented before dental colleges had seriously taken up this study, and, doubtless, had much to do with forming professional opinion as to its value as a dental study. It became the pioneer in this work and an incentive to others to write in the same direction. At present all the dental colleges of this country make some attempts to supply the needs of students by establishing metallurgy as part of the curriculum, and some possess elaborate facilities for teaching this very important sub-branch of prosthetic dentistry.

While the foregoing is true of the book as a whole, there are some of the subjects that might be improved by greater elaboration and more attention to detail, and also would have been greatly improved by illustrations. Allusion is here made to the refining of gold and silver. The process is fully stated and sufficiently so for one practically familiar with the methods used, but it is thought that one untrained in the work would be greatly at a loss to follow the instruction as given. The student needs to be shown the process step by step. In illustration, if he is required to refine scraps or filings, the process should be followed from the first removal of iron from the mass until it finally reaches the ingot from the crucible. The processes detailed are all correct, but lack this special attention to details which marks the experienced teacher when dealing with inexperience.

The book is enriched through the labor of Dr. Augustus Koenig, and also through valuable suggestions by his father, Professor George A. Koenig, M.E., Ph.D., Professor of Chemistry and Metallurgy in the Michigan College of Mines.

Discovery of Anæsthesia by Horace Wells. Memorial Services at the Fiftieth Anniversary. Patterson & White Company, Philadelphia, 1900.

It may seem strange that six years have been permitted to elapse since the celebration of the discovery of anæsthesia took place, December 11, 1894, before this book was permitted to see the light; but it was regarded by the committee having in charge the preparation of the bust of Horace Wells that the published report should be supplementary to its completion and installation in the Library of the Medical Museum at Washington. The preparation of this consumed more time than was originally anticipated, hence the delay in the appearance of this memorial volume. The dental profession is generally familiar with the proceedings connected with that celebration, through the reports in the dental journals at the time, but as part of the history of this the greatest discovery of the nineteenth century, no dentist can afford to neglect this opportunity to add this memorial volume to his library.

The two principal addresses of that day were that of Professor Fillebrown, of Boston, upon the History of Anæsthesia, a carefully prepared and exhaustive paper and worthy of permanent preservation, and that by Professor James E. Garretson upon Horace Wells.

There is an interest attached to the latter, as it was the last of Dr. Garretson's public efforts, and it seems to the writer to have been one of the most brilliant productions of this remarkable man. It was a tribute from the great surgeon to the man who had made modern surgery possible. Garretson has passed beyond the activities of earth, but he has left us this address, and, perhaps, the most eloquent and vivid description in the English language of the difference in the amount of suffering before the days of anæsthesia and the relief from pain which followed.

After describing an operation performed by him the day previous for the removal of a tumor which involved the salivary glands, trachea, carotid arteries, jugular vein, and pneumogastric nerves, while the patient was "sleeping and dreaming quietly," he continued: "Consider, in contrast, a picture familiar before the days of Wells's inspiration. A mother, her heart welling out in tears, limbs trembling so as scarcely to afford her support, helpless misery marking her countenance, despair striking at her with its thongs of flame, follows into a hospital operating-amphitheatre a nurse who carries her first-born, which is being brought to the table. Alas! helpless, indeed, is the mother. How more gladly, how a thousand times more than gladly, would she lie down in place of the child. Cries of mother and child moan through the hospital, and the least sensitive feels his cheek pale. The crucial moment has come. The child is placed and held by force upon the table. The mother is torn away. For a single moment eyes of mother and child have met in parting. A loud, frightened, despairing cry from the child rings from ceiling to floor of the room. The mother drops in a heap and is carried out a raving lunatic. She raves about and curses God as being without pity or mercy.

"Let a picture of to-day have relation with that other one of the past. One which extended, alas! from the days of the first surgical performance to the year of grace eighteen hundred and forty-four.

"A mother brings to a hospital a child whose deformity requires the knife for its correction. Conscious of the power of anæsthesia, the surgeon talks to the parent, while all the while the little patient, pleased and inveigled by the sweet smell of chloroform, is itself anæsthetizing itself. The cutting is done. The child has a dream of roses and gardens and wide fields. The mother has placed in her arms her restored offspring. She has no tears, no words; her contact has been alone with beneficence. She is overwhelmed by the mystery met and passed. She says, 'Our Father which art in heaven.' She says and feels there is a God of pity and mercy.

"Look at the name of the maker of these pictures of the new time! It reads, Horace Wells."

The publishers of this volume are to be congratulated upon the tasteful character of the book. It is in every respect worthy the subject.

The presentation of this memorial volume closes the labors of the committee, and it is regarded as a fitting and appropriate tribute from the dentists of America to one of the great benefactors of the human race developed in this century,—Horace Wells.

An excellent reproduction of the bust of Horace Wells is placed as a frontispiece to the volume.

Those desiring the book can procure it by enclosing one dollar to Patterson & White Company, 518 Ludlow Street, Philadelphia, Pa.

